# Biomarker and Histopathology Evaluation of Patients with Recurrent Glioblastoma Treated with Galunisertib, Lomustine, or the Combination of Galunisertib and Lomustine

**DOI:** 10.3390/ijms18050995

**Published:** 2017-05-06

**Authors:** David Capper, Andreas von Deimling, Alba A. Brandes, Antoine F. Carpentier, Santosh Kesari, Juan M. Sepulveda-Sanchez, Helen R. Wheeler, Olivier Chinot, Lawrence Cher, Joachim P. Steinbach, Pol Specenier, Jordi Rodon, Ann Cleverly, Claire Smith, Ivelina Gueorguieva, Colin Miles, Susan C. Guba, Durisala Desaiah, Shawn T. Estrem, Michael M. Lahn, Wolfgang Wick

**Affiliations:** 1 Department of Neuropathology, Charité Universitätsmedizin Berlin, Charitéplatz 1, 10117 Berlin, Germany; 2 Department of Neuropathology, University Hospital Heidelberg and Clinical Cooperation Unit Neuropathology, German Cancer Consortium (DKTK), German Cancer Research Center (DKFZ), 69120 Heidelberg, Germany; andreas.vondeimling@med.uni-heidelberg.de; 3 Medical Oncology Department, Bellaria-Maggiore Hospitals, Azienda USL—IRCCS Institute of Neurological Sciences, 40139 Bologna, Italy; a.brandes@ausl.bo.it; 4 Assistance Publique-Hôpitaux de Paris (AP-HP) & Paris 13 University, Hôpital Avicenne, Service de Neurologie, 93009 Bobigny, France; antoine.carpentier@avc.aphp.fr; 5 UC San Diego Health System, La Jolla, CA 92103, USA; kesaris@jwci.org; 6 Hospital Universitario 12 de Octubre, 28041 Madrid, Spain; jmsepulveda.hdoc@salud.madrid.org; 7 Department of Oncology, Royal North Shore Hospital, St. Leonards, NSW 2065, Australia; hrwheeler@optusnet.com.au; 8 CHU Hôpital De La Timone, Rue Saint Pierre, 13385 Marseille, France; olivier.chinot@ap-hm.fr; 9 Austin Hospital, Heidelberg, VIC 3084, Australia; lmcher@mac.com; 10 Dr. Senckenberg Institute of Neurooncology, University Hospital Frankfurt, 60590 Frankfurt, Germany; Joachim.steinbach@med.uni-frankfurt.de; 11 Antwerp University Hospital, Wilrijkstraat 10, 2650 Edegem, Belgium; pol.specenier@uza.be; 12 Medical Oncology, Vall d’Hebron University Hospital, Calle Natzaret, 115-117, 08035 Barcelona, Spain; jrodon@vhebron.net; 13 Eli Lilly and Company, Erl Wood Manor, Windlesham GU20 6PH, UK; cleverly_ann_louise@lilly.com (A.C.); csmith@lilly.com (C.S.); gueorguieva_ivelina@lilly.com (I.G.); miles_colin_p@lilly.com (C.M.); 14 Eli Lilly and Company, Indianapolis, IN 46285, USA; guba_susan_c@lilly.com (S.C.G.); desaiah_durisala@lilly.com (D.D.); estrem_shawn_t@lilly.com (S.T.E.); michalahn@aol.com (M.M.L.); 15 Department of Neurology, University Hospital Heidelberg, 69120 Heidelberg, Germany; wolfgang.wick@med.uni-heidelberg.de

**Keywords:** galunisertib monohydrate (LY2157299), TGF-β, pSMAD2, CDK4/CDK6, biomarkers

## Abstract

Galunisertib, a Transforming growth factor-βRI (TGF-βRI) kinase inhibitor, blocks TGF-β-mediated tumor growth in glioblastoma. In a three-arm study of galunisertib (300 mg/day) monotherapy (intermittent dosing; each cycle =14 days on/14 days off), lomustine monotherapy, and galunisertib plus lomustine therapy, baseline tumor tissue was evaluated to identify markers associated with tumor stage (e.g., histopathology, Ki67, glial fibrillary acidic protein) and TGF-β-related signaling (e.g., pSMAD2). Other pharmacodynamic assessments included chemokine, cytokine, and T cell subsets alterations. 158 patients were randomized to galunisertib plus lomustine (*n* = 79), galunisertib (*n* = 39) and placebo+lomustine (*n* = 40). In 127 of these patients, tissue was adequate for central pathology review and biomarker work. Isocitrate dehydrogenase (*IDH1*) negative glioblastoma patients with baseline pSMAD2^+^ in cytoplasm had median overall survival (OS) 9.5 months vs. 6.9 months for patients with no tumor pSMAD2 expression (*p* = 0.4574). Eight patients were IDH1 R132H^+^ and had a median OS of 10.4 months compared to 6.9 months for patients with negative IDH1 R132H (*p* = 0.5452). IDH1 status was associated with numerically higher plasma macrophage-derived chemokine (MDC/CCL22), higher whole blood FOXP3, and reduced tumor CD3^+^ T cell counts. Compared to the baseline, treatment with galunisertib monotherapy preserved CD4^+^ T cell counts, eosinophils, lymphocytes, and the CD4/CD8 ratio. The T-regulatory cell compartment was associated with better OS with MDC/CCL22 as a prominent prognostic marker.

## 1. Introduction

Glioblastoma is the most common brain cancer in adults, and, despite aggressive treatment with surgery and chemoradiation, the median survival remains approximately 15 months from initial diagnosis [1]. Glioblastoma is genetically and histopathologically diverse, which may require the development of inhibitors for specific subgroups of patients [2,3,4,5]. Transforming growth factor-β (TGF-β) signaling is a pathway that appears to be active in a specific subgroup of patients with glioblastoma [6,7,8,9,10,11]. For example, glioma cells that escape vascular endothelial growth factor (VEGF) inhibition continue to grow in a TGF-β-dependent manner [6]. Also, elevated TGF-β plasma ligand levels may be associated with increased numbers of T-regulatory cells in patients with glioblastoma [7]. The presence of both active TGF-β signaling and T-regulatory cells is hypothesized to shorten the prognosis of glioma patients, especially if TGF-β2 is expressed [8]. Upon engagement of the TGF-β receptor complex, the intracellular kinase of SMAD2 is activated by phosphorylation [8]. Hence, pSMAD2 expression in tumor tissue is commonly used to describe the degree of TGF-β signaling activation. pSMAD2 can be found either in a cytoplasmic or in a nuclear form, and nuclear pSMAD2 is considered the best read-out for a condition with activated TGF-β signaling. When using pSMAD2 as a marker for TGF-β signaling activation in glioblastoma patients, it appears that patients with high pSMAD2 expression have more aggressive and treatment-resistant tumors associated with low overall survival (OS) [9,10,11]. Thus, inhibitors of TGF-β signaling were postulated to improve OS by modifying tumor cell growth and restoring anti-tumor immunity.

The small molecule inhibitor (SMI) galunisertib (LY2157299 monohydrate) showed anti-tumor effects in glioblastoma animal models and also in patients [10,11,12,13,14,15]. Galunisertib targets the serine-threonine kinase of the TGF-βRI and abrogates the phosphorylation of SMAD2, the initial intracellular protein of the TGF-β signaling pathway [12]. The overall activity of galunisertib was further supported by tumor responses in 16% of patients who progressed on their first- and second-line treatments for glioblastoma [13,14]. Preclinical studies also suggested that the combination of lomustine and galunisertib may have synergistic, or at least additive, anti-tumor effects [15]. Given the scientific hypothesis of blocking the TGF-β signaling pathway and based on the observations of preclinical and clinical anti-tumor activity in patients with glioblastoma, a Phase 2 study was initiated to evaluate the anti-tumor activity, safety, pharmacokinetics, and biomarker activity of galunisertib in combination with lomustine in patients with recurrent glioblastoma [16]. Unfortunately, the study did not show clinical benefit for galunisertib in combination with lomustine or galunisertib monotherapy.

Here we report on the correlative biomarker studies conducted during the Phase 2 study [16]. Using a central neuropathology evaluation of all tumor tissue samples, it was possible to evaluate novel biomarkers related to TGF-β signaling in addition to a histopathology examination.

## 2. Results

### 2.1. Outcome from the Tissue Collection

In this three-arm study, 158 patients were enrolled [16] of whom 142 patients had tumor tissue for central pathology review (90%), including 15 patients with unacceptable quality. In total, tumor tissue from 127 patients was considered to be of acceptable quality (127/158; 80%). Among the 127 tumor specimens, 118 were collected within two years of enrollment (Table S1).

### 2.2. Description of Standard Pathology Evaluation

Of the 127 patients with evaluable tissues, 120 patients had a confirmed grade IV diagnosis, including one case with a giant cell glioblastoma and three with gliosarcoma (Table 1).

Based on the most recent diagnostic tumor specimens, the congruence between local neuropathologist and central review was over 90% (Table S2).

Most patients had medium cellular density (*n* = 100), endothelial hypertrophy (*n* = 124), and necrosis without pseudopalisading (*n* = 72) (Table 1). Approximately 20% of patients had presence of small cell astrocytoma-like foci (*n* = 27) and microcysts (*n* = 24). Most tumor cells had medium nuclear abnormalities (*n* = 92) and 6–20 mitoses (*n* = 66). Ki67 staining in 11–20% of cells was seen in 43 specimens, while the median H score for GFAP was 140. IDH1 R132H mutation was detected in eight specimens. No methylguanine methyltransferase (MGMT) assessment was conducted in this study. In all evaluable specimens, immunohistochemistry (IHC) detected pSMAD2 in the nuclei and in 22 cases (22/127; 17.3%) in the cytoplasm. CD3 IHC was assessed in two anatomical locations; perivascular and diffuse parenchymal infiltrates (Table 1). In 20 cases, CD3^+^ cell infiltrates were detected in the perivascular compartment with T cell infiltration, constituting ≥5% of the total parenchymal cell. Cellular density and the number of mitoses were correlated, and each was also correlated with Ki67 staining (Supplemental Table S3). In contrast, CD3 staining was not correlated with cellular density, mitoses, or Ki67 (data not shown).

### 2.3. Association of Histopathology and Overall Survival (OS)

**Figure 1:**
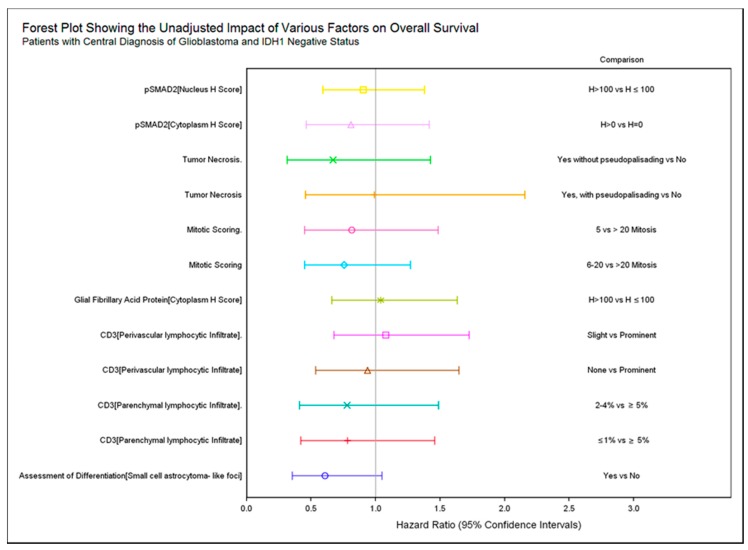
Forest plot showing the univariate impact of various tissue markers on overall survival (OS). HR < 1 indicates improved OS for the left hand side of the comparison compared to the right hand side. For example, patients with a pSMAD2 Nucleus H score >100 had numerically longer OS than patients with a pSMAD2 Nucleus H score ≤100. No comparisons reached statistical significance.

We examined whether the histopathological findings from the original diagnostic tissue correlated with OS amongst patients with IDH1 negative glioblastoma. No statistically significant associations with OS were observed for any of the histological features summarized in Table 1 (Figure 1).

**Figure 2:**
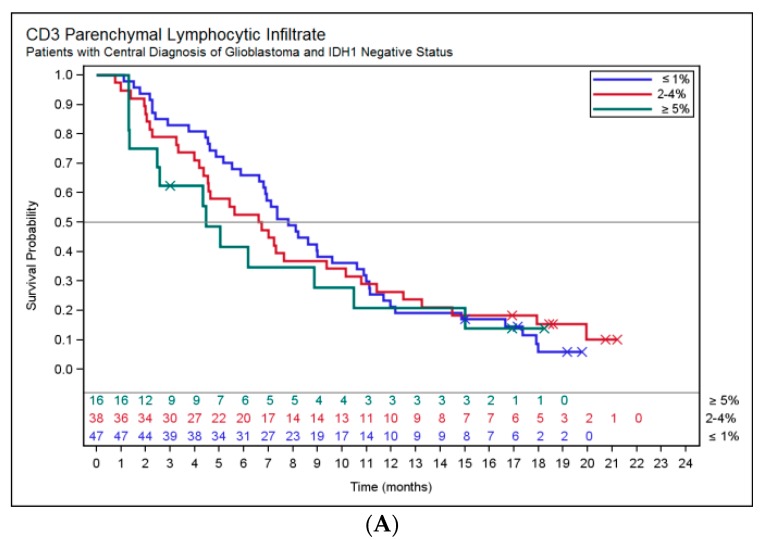

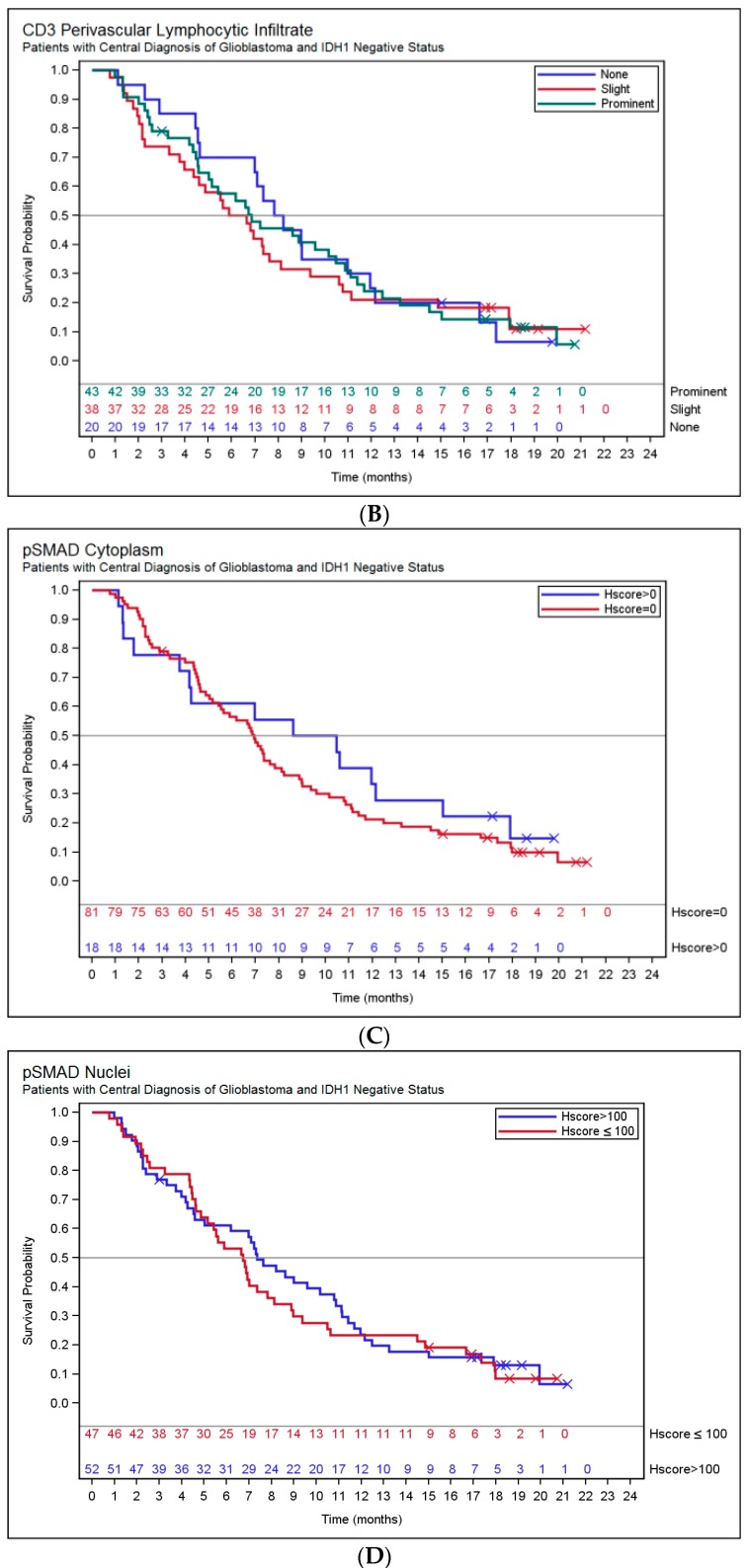
(**A**–**D**): Kaplan–Meier plots showing the impact of tissue CD3 and pSMAD2 on OS. No comparisons reached statistical significance. Respective log rank *p*-values are: (**A**) 0.2169; (**B**) 0.9360; (**C**) 0.2076; and (**D**) 0.4499.

While not statistically significant, patients with increased parenchymal CD3^+^ lymphocytic infiltrate seemed to have shorter OS: ≤1% median OS 7.8 months; 2–4% median OS 6.7 months; ≥5% median OS 4.5 months (log rank *p*-value 0.7111) (Figure 2A,B).

When the data for the level of CD3^+^ infiltrates in both the vascular and ≥5% parenchymal compartments were combined, the median OS was 4.5 months compared to 7.2 months for all other patients with no or reduced CD3^+^ T cell infiltrates (log rank *p*-value = 0.1668) (data not shown). CD3^+^ cell infiltration in the tumor tissue was not significantly associated with peripheral lymphocyte counts, TGF-β1 plasma levels, macrophage-derived chemokine (MDC/CCL22) levels, or blood CD3% assessed at study enrollment (Table S4a).

Patients with positive staining for cytoplasmic pSMAD2 had a median OS of 9.5 months compared to patients with no cytoplasmic pSMAD2, who had a median OS of 6.9 months (log rank *p*-value 0.4574) (Figure 2C,D).

No association was found between tissue pSMAD2 expression and TGF-β1 levels in plasma, FOXP3 in blood, or CD4^+^CD25^+^CD127^−^/LOFOXP3^+^ in plasma, collected at the time of study enrollment (Table S4b). In order to verify if temporal changes in parameters may be impacting the ability to determine associations between tissue and plasma markers, a sensitivity analysis including only the 36 patients with tissue collected within two months of study enrollment was conducted, and still no associations were found (data not shown).

### 2.4. IDH1 Subgroup Analysis

TGF-β-related signaling protein pSMAD2 was not increased in IDH1 mutation-positive patients (Table 2).

Tissue CD3^+^ staining was greater in patients with IDH1 mutation-negative tumors compared to patients with IDH1 mutation-positive tumors (*p* = 0.0260). Also, plasma levels of FOXP3 were higher in IDH1-positive patients (*p* = 0.0394) (Table 2). Additionally, plasma levels of MDC/CCL22, TGF-β1, and CD4^+^ T cells were numerically higher in IDH1 positive patients compared to IDH1 negative patients (not statistically significant).

### 2.5. Pharmacodynamic Responses

Post-treatment changes relative to baseline values were assessed for the following parameters; CD4^+^, CD8^+^, the ratio of CD4^+^/CD8^+^, CD4^+^CD25^+^CD127^−^/LOFOXP3^+^, FOXP3 (%), and CD3 (%) by whole blood assay, eosinophils, lymphocytes, neutrophils, the neutrophil/lymphocyte ratio, monocytes, lactate dehydrogenase (LDH), S100β, C-reactive protein (CRP), MDC/CCL22, and TGF-β1 in plasma.

#### 2.5.1. Immune Monitoring and T Cell Subsets by Flow Cytometry

At baseline, blood CD4^+^ T cell counts and CD4^+^CD25^+^CD127^−^/LOFOXP3^+^ T cell subsets were generally low. By contrast, CD8^+^ T cell subsets were mostly normal.

**Figure 3:**
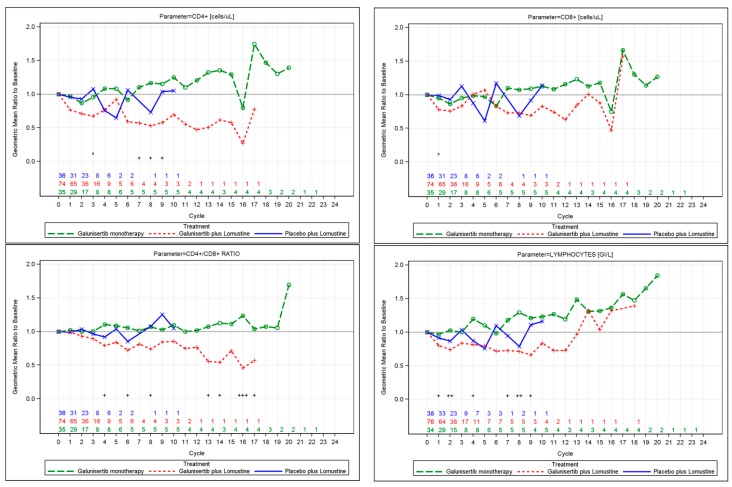

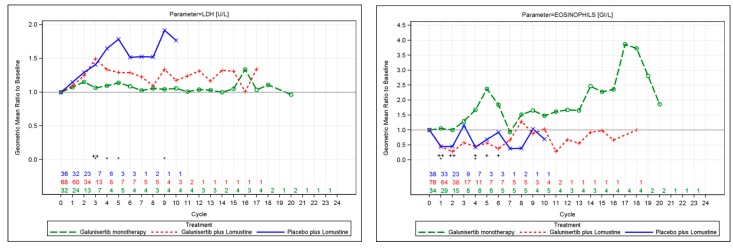
Line plot of least-square (LS) mean fold change in laboratory markers over time by treatment. Numbers annotated on the plot represent the number of patients with evaluable data for each cycle. Results need to be interpreted with caution since sample sizes are small at later cycles as patients discontinued treatment. The following parameters had an overall treatment effect of *p* < 0.05: CD4^+^, CD4^+^/CD8^+^ ratio, lymphocytes, lactate dehydrogenase (LDH), eosinophils. Additional parameters that were considered in exploratory analyses included serum C-reactive protein (CRP), blood CD3^+^, FOXP3, CD8^+^, CD4^+^CD25^+^CD127^−^/LOFOXP3^+^, monocytes, neutrophils, neutrophil/lymphocyte ratio, and TGF-β1, but no consistent significant differences among treatment arms were observed over time; overall treatment *p* > 0.05. Comparison of galunisertib monotherapy versus placebo plus lomustine, * *p* < 0.05, ** *p* < 0.01, *** *p* < 0.001; Comparison of galunisertib monotherapy versus galunisertib plus lomustine, + *p* < 0.05, ++ *p* < 0.01; +++ *p* < 0.001; Comparison of galunisertib plus lomustine versus placebo plus lomustine, ^ *p* < 0.05, ^^ *p* < 0.01, ^^^ *p* < 0.001.

While on treatment, there were significant differences in CD4^+^, CD4^+^/CD8^+^ ratio, eosinophils, and total lymphocyte counts. All these immune cells were reduced in patients treated in both lomustine-containing arms compared to the galunisertib monotherapy arm (overall treatment *p* < 0.05). Compared to the lomustine-containing treatments, eosinophils, neutrophils, total lymphocyte, and monocyte counts remained largely unchanged during monotherapy with galunisertib. Differences in CD4^+^ T cell counts and CD4^+^/CD8^+^ ratio were more apparent from cycle 4 onward, but this observation is limited because most patients discontinued treatment at cycle 2 (Figure 3).

For each of the five laboratory markers, patients with evaluable cycle 2 assessments were classified as having increased or decreased changes from baseline at cycle 2 (fold change ≥1 vs. fold change <1). Increased values of each of the laboratory markers at cycle 2 were not prognostic for OS (*p* > 0.05) for any parameter in univariate analyses using Cox regression models.

Other ratios such as CD8/CD3 (%) or CD8/total lymphocyte counts were explored, but there were no changes observed in these subpopulations (data not shown).

#### 2.5.2. Plasma Markers

TGF-β1 levels were not changed over time (data not shown). MDC/CCL22 levels were measured at baseline and at cycle 2. Mean reductions from baseline in MDC at cycle 2 were 11% (95% CI: −14% to 31%) for the galunisertib monotherapy arm, 27% (95% CI 13% to 39%) for the galunisertib plus lomustine arm, and 37% (95% CI 20% to 51%) for the placebo plus lomustine arm (data not shown). We also measured plasma levels of interleukin-2 (IL-2) using the same multi-analyte panel as for MDC/CCL22 levels, but the levels for IL-2 were undetectable (data not shown).

LDH had a greater increase for the two lomustine arms relative to the galunisertib monotherapy arm from cycle 3 onward, noting that interpretation is limited given the small sample sizes remaining in the study at later visits (data not shown).

None of the other parameters listed demonstrated significant differences in the changes from baseline among the treatment arms overall.

#### 2.5.3. Genetic Evaluation

Genetic variants (substitutions, short insertions and deletions, and copy number alterations) across 287 cancer related genes were identified for 70 tumor samples (~45% of the patients). The number of known/likely functional mutations detected per patient ranged from 1 to 12. The genes and types of mutations observed in this glioblastoma population matched the frequencies observed in previously characterized glioblastoma populations [17]. For example, epidermal growth factor receptor (*EGFR*) amplification/mutation was observed in 32 tumors (46%), *CDKN2A* deletion detected in 39 (56%), and cyclin-dependent kinase-4 (*CDK4*) amplification in 10 (14%). However, in contrast with previous observations in a smaller galunisertib-treated glioblastoma patient population [13], genetic variants in these three genes were identified in both galunisertib responsive and non-responsive tumors.

Previously, four of five *IDH1* mutated tumors benefitted from galunisertib treatment [13]. Here, five of the sequenced tumors contained an IDH1 mutation. These five tumors were also determined to be *IDH1* mutated by IDH1 R132H IHC assessment, and an additional three *IDH1* mutated tumors were identified by IHC (sequence data not available).

## 3. Discussion

Although we observed no treatment differences among the three treatment arms [16], we here present the integration of tissue- and blood-based biomarker examination in the same study. With the present approach, we assessed tumor tissue with histopathology and compared them to plasma markers. Plasma markers also allowed serial measurements of pharmacological changes in each treatment arm. The overall assessment, conducted centrally by neuropatholgists and central laboratories, including T cell subsets, is one of the most detailed assessments in glioblastoma patients treated with a TGF-β inhibitor. Despite the lack of treatment differences between the three arms, we here report also on some trends that may be useful to evaluate in future studies investigating TGF-β inhibition.

Compared to the histopathology results provided by local histopathology laboratories, the results of the central review deviated in only seven patients from the local diagnosis of glioblastoma (Table S2). Overall, the standard histopathology review was consistent with a typical glioblastoma patient population with a low number of IDH1 R132H^+^ tumor specimens [18]. The anatomical features associated with Ki67, such as mitoses and cellular density, were correlated (Table S3). MGMT status was not part of the assessment but should be included for studies in which temozolomide-based therapy is part of the clinical investigation [19]. The reason for not performing MGMT testing was based on the limited amount of tumor tissue and the focus on TGF-β-associated signaling and immune markers. Today, advanced technology allows for MGMT testing with smaller tumor tissue specimens; hence, this test may be integrated in future TGF-β-directed studies.

As previously reported for other studies with galunisertib [13,16], high baseline plasma levels of MDC/CCL22 were associated with better OS. In addition, we found that blood CD4^+^ T cell counts, T regulatory cells, and FOXP3 levels were also correlated with improved OS. In tumor tissue, the presence of CD3^+^ T cells in the parenchyma appeared to be numerically associated with reduced survival but were not statistically significant. Interestingly, there was no correlation between the CD3 levels as determined by an epigenetic test in blood and the CD3^+^ presence in tumor tissue as determined by IHC. The association of tumor-infiltrating CD3^+^ T cells with poor survival is different from other reports in which CD3^+^ tumor-infiltrating lymphocytes (TILs) are associated with improved OS, such as colorectal [20,21]. However, for glioblastoma, the association between CD3^+^ TILs and OS is less clear and mostly reported as inconclusive [22,23,24].

We also attempted to stain for the presence of CCL22 in tumor tissue but were not able to establish a satisfactory staining protocol, which was based on previous work on lymphoma tissue [25]. Thus, it is not clear whether the high levels of CCL22 originate from the tumor or are produced systemically. This is important information as CCL22 may be produced by IDO^+^ dendritic cells in glioblastoma, which in turn may increase the generation of T regulatory cells in glioblastomas [26]. Interestingly, we also observed that patients with IDH1 R132H^+^ tumors had different immune baseline characteristics. These patients tended to have higher levels of markers associated with T regulatory cells.

The TGF-β-related pathway in tumor tissue was assessed by staining for pSMAD2 as a marker of pathway activation. Similar to the First-in-Human Dose study [13], we observed that patients with higher baseline pSMAD2 levels in the tumor tissue had numerically better survival, but this was not statistically significant. Previous studies, however, found that high pSMAD2 staining was associated with poor survival [9], and perhaps these discrepancies may be explained by differences in staining protocols, assessment of cellular compartment (cytoplasmic versus nuclear staining), stage of tumor, and time interval since initial diagnosis. A limitation for this evaluation, however, represents the time interval of study participation and the pSMAD2 assessment on the original diagnostic tumor tissue. Generally, the time interval was about one year since the initial tumor was obtained and the patient started in this study. However, in this period, pSMAD2 expression may have changed, especially because patients completed chemo-radiation, which in turn may have affected the pathway activation.

Considering the other biomarker observations, such as correlation for better outcome with T regulatory like conditions (e.g., MDC/CCL22 levels, presence of T regulator cells), it appears as if T regulatory cells have a possible benefit in glioblastoma patients.

In accordance with previous reports, there was a trend toward positive correlation between IDH1 and OS. This trend was not statistically significant, possibly due to the small sample of IDH1-positive tumors. Gene mutations were also assessed in a subgroup of patients from which tumor material was left from the original investigation. In this subgroup of patients, we observed concordance (5/5) of the IDH1 R132H staining and DNA sequencing methods.

Across all treatment arms, plasma levels of TGF-β1 were reduced with no difference among the treatments (data not shown). In other studies, reductions of plasma TGF-β1 levels were associated with either the removal of tumor mass after surgery [27] or responses after chemotherapy [28].

However, there were some trends that are worth mentioning so they can be prospectively tested in future trials. First, lomustine treatments (with and without galunisertib) appeared to affect the lymphocyte counts. Second, patients who received only galunisertib seemed to conserve their lymphocyte counts and their subsets (Figure 3). Among these patients there was a small subgroup that benefited from longer treatment with galunisertib and showed an increase in CD4^+^ T cell and lymphocyte counts. In contrast to the galunisertib monotherapy, for patients on the other two treatments containing lomustine, CD4^+^ T cell and lymphocyte counts seemed to decrease over time. Since the CD4/CD8 ratio did not change and lymphocytes increased overall, one should evaluate other lymphocyte subsets in future studies in galunisertib-treated patients. Third, patients who maintained their baseline levels of lymphocyte counts showed no increases of LDH (Figure 3).

In conclusion, our data support earlier studies, indicating that glioblastoma patients with conditions supporting T regulator cells have an improved survival rate [29]. The pre-existing conditions that may pre-dispose patients to develop glioblastoma may be similar to patients with increased peripheral immune tolerance and subsequent higher incidences of lung, colorectal, and estrogen receptor (ER)-negative breast cancer [30].

## 4. Materials and Methods

### 4.1. Patients

Adult male and female patients ≥18 years of age were eligible for enrollment in the study if they had been diagnosed with recurrent intracranial glioblastoma (World Health Organization Grade IV) confirmed by histological evaluation [3]. All patients had to have ≤1 on the Eastern Cooperative Oncology Group (ECOG) performance status scale. Patients were required to have evidence of tumor progression as determined by Response Assessment in Neuro Oncology (RANO) criteria following standard chemoradiation [1]. The study was conducted according to the principles of good clinical practice, applicable laws and regulations, and the Declaration of Helsinki. Each institution’s review board approved the study, and all patients signed an informed consent document before study participation. Informed consent was obtained from all individual participants included in the parent study from which the tissue and blood/plasma samples were obtained for this analysis.

### 4.2. Study Design

This was a three-arm, randomized, multinational, Phase 2 study of galunisertib monotherapy or galunisertib plus lomustine compared to lomustine plus placebo in patients with relapsed or progressed glioblastoma (Clinical Trial Registration: NCT01582269, 19 April 2012, ClinicalTrials.gov). Eligible patients were randomized in a 1:2:1 manner to these three treatment arms [16]. As previously described, anti-tumor activity was primarily based on assessing OS, and the secondary endpoints included overall response rate (ORR) based on RANO criteria [16].

#### 4.2.1. Central Pathology Review and Immunohistochemistry

Formalin-fixed paraffin-embedded tumor specimens from the original diagnostic tissue were obtained and 5-μm sections were prepared. In cases in which a patient had more than one tumor tissue sample, the most recent diagnostic specimen was used for the central pathology review. The neuropathologist was blinded to treatments and their outcome and performed the subsequent examinations in a blinded fashion. All data were entered in a standardized fashion into a database and later combined with the clinical data.

Hematoxylin and Eosin (H&E) staining was performed to assess the general anatomical phenotype using characteristics such as differentiation, cellular density, vessel structure, tumor necrosis, nuclear abnormalities, and mitotic scoring.

Additional staining was performed to assess the expression of glial fibrillary acidic protein (GFAP), Ki67, isocitrate dehydrogenase 1 (IDH1), R132H, pSMAD2, and CD3. Furthermore, tumor specimens were stained for MDC/CCL22 [25]. The process of evaluation was based on the standard IHC staining developed at the neuropathology laboratory at the University Clinic of Heidelberg, Heidelberg, Germany [18].

#### 4.2.2. Central Laboratory Evaluation for Blood Based Markers

Blood laboratory parameters included blood CD3 (%) (normal range: 17% to 36%), FOXP3 (%) (normal range: 1% to 3.6%), CD4^+^ (normal range: 441 to 2156 cells/μL), CD8^+^(normal range: 125 to 1312 cells/μL), CD4^+^CD25^+^CD127^−^/LOFOXP3^+^ (normal range estimated to be: approximately 18 to 86 cells/μL), neutrophils (normal range: 2.03 to 8.36 GI/L), lymphocytes (normal range: 1.02 to 3.36 GI/L), monocytes (normal range: 0.16 to 0.91 GI/L), eosinophils (normal range: 0 to 0.56 GI/L), plasma macrophage-derived chemokine/chemokine (c–c motif) ligand-22 (MDC/CCL22) (normal range: 181 to 571 pg/mL), plasma TGF-β1 (normal range: 741 to 3472 pg/mL), serum S100β (normal range: 0 to 96 ng/L), serum LDH (normal range: 80 to 250 U/L), and serum C-reactive protein (high sensitivity) (normal range: 0 to 3 mg/L).

Plasma samples from patients were analyzed for TGF-β1 levels by enzyme‑linked immunosorbent assay (ELISA) (R&D Systems, DB100B, Minneapolis, MN, USA). MDC/CCL22 levels were determined at baseline using the multi-analyte immunoassay panel (MAIP) developed by Myriad/RBM (Austin, TX, USA). Whole blood samples were used to determine the levels of T cell subsets such as CD4^+^ and CD8^+^ and CD4^+^CD25^+^CD127^−^/LOFOXP3^+^ by standard flow cytometry. In addition, the percentages of FOXP3 and CD3 were determined in whole blood using an epigenetic T cell assay (Epiontis, Berlin, Germany) [31].

#### 4.2.3. Genetic Evaluation

For 70 patients, there was sufficient tumor tissue from which DNA for mutation assessments was extracted and sequenced for 287 frequently mutated cancer genes by Foundation Medicine, Cambridge, MA, USA [32]. The 70 patients were distributed across the three arms of the study and were representative of the intent-to-treat (ITT) population.

### 4.3. Statistical Analyses

Potential prognostic histopathology factors, as measured at baseline, were evaluated for their impact on OS, utilizing univariate Cox models from which any characteristics with *p* ≤ 0.05 were to be selected for subsequent multivariate analysis. In addition, the OS for patients with CD3^+^ lymphocytic infiltration and OS for patients with positive staining for pSMAD2 were summarized descriptively using the Kaplan–Meier method. Associations between tissue and plasma markers were summarized; *p*-values for comparisons were calculated by analysis of variance using log transformed data for continuous measures and Fisher’s exact test for categorical measures. Post-treatment changes relative to baseline values were evaluated for laboratory markers. Data were log_e_-transformed prior to analysis, and the log ratio to baseline was analyzed by mixed effect model repeated measures (MMRM), adjusting for treatment, visit, treatment-by-visit interaction, and baseline-by-visit interaction. Repeated assessments were accounted for using an unstructured variance covariance matrix. For parameters with an overall treatment effect of *p* ≤ 0.05, the geometric mean ratio to baseline was presented for each treatment group.

## Figures and Tables

**Table 1:** Summary of histopathological evaluation.

Evaluation	Result	All Patients *n* (%) ^a^	Patients with Glioblastoma, Gliosarcoma or Giant Cell Glioblastoma, and IDH1 R132H Negative *n* (%) ^a^
Diagnosis	Glioblastoma	116 (92)	103 (96)
	Glioma, astrocytoma	3 (2)	0
	Glioma, gliosarcoma	3 (2)	3 (3)
	Glioma, pleomorphic xanthoastrocytoma	1 (1)	0
	Protoplasmic astrocytoma	1 (1)	0
	Oligoastrocytoma	1 (1)	0
	Giant cell glioblastoma	1 (1)	1 (1)
Assessment of differentiation	Perinuclear halos	11 (9)	7 (7)
	Fibrillary astrocytoma-like foci	9 (7)	6 (6)
	Small cell astrocytoma-like foci	27 (21)	21 (20)
	Polar spongioblastic foci	0	0
	Protoplasmic astrocytoma-like foci	1 (1)	0
	Minigemistocytes	5 (4)	2 (2)
	Classic gemistocytes	16 (13)	12 (11)
	Giant cells	16 (13)	15 (14)
	PNET-like	13 (10)	11 (10)
	Sarcoma-like	7 (6)	7 (7)
	Microcysts	24 (19)	18 (17)
	Mucoid degeneration	11 (9)	8 (8)
	Calcifications	2 (2)	2 (2)
Vessel structure	Abnormal number of vessels	117 (93)	101 (94)
	Any endothelial hypertrophy	124 (100)	107 (100)
	Glomeruloid blood vessel	69 (56)	62 (58)
	Multi-layering blood vessel	116 (94)	103 (96)
	Vascular abnormalities	124 (98)	107 (100)
	Vessel thrombosis	87 (69)	80 (75)
Cellular density	Low (like diffuse astrocytoma)	5 (4)	5 (5)
	Medium (like classical glioblastoma)	100 (79)	86 (80)
	High (like PNET)	21 (17)	16 (15)
Tumor necrosis	Yes, with pseudopalisading	36 (29)	34 (32)
	Yes, without pseudopalisading	72 (57)	64 (60)
	No	18 (14)	9 (8)
Mitotic scoring	≤5 Mitoses (per 10 Highpower fields)	31 (25)	24 (23)
	6–20 Mitoses (per 10 Highpower fields)	66 (53)	59 (56)
	>20 Mitoses (per 10 Highpower fields)	28 (22)	23 (22)
Nuclear abnormalities	Low (nuclear aspect as in normal glial cells)	4 (3)	1 (1)
	Medium (abnormal nuclear shape)	92 (73)	78 (73)
	High (bizarre nuclei)	30 (24)	28 (26)
CD3 parenchymal lymphocytic infiltrate	≤1%	54 (47)	47 (45)
	2–4%	41 (36)	40 (38)
	≥5%	20 (17)	18 (17)
CD3 perivascular lymphocytic infiltrate	None (≤4 perivascular positive cells per vessel)	24 (21)	21 (20)
	Slight (≥1 vessel with ≥5 and <30 positive perivascular cells)	43 (37)	38 (36)
	Prominent (≥1 vessel with ≥5 and ≥30 positive perivascular cells)	48 (42)	46 (44)
IDH1 R132H	Positive	8 (7)	0
	Negative	108 (93)	107 (100)
Ki67	≤5%	6 (6)	6 (6)
	6–10%	25 (24)	23 (24)
	11–20%	43 (41)	37 (39)
	>20%	32 (30)	30 (31)
Glial fibrillary acid protein	Cytoplasm Total Detected (H score > 0)	124 (100)	105 (100)
	Cytoplasm H score Median (25th percentile, 75th percentile)	140 (70, 210)	150 (90 210)
pSMAD2	Cytoplasm Total Detected (H score > 0)	22 (18)	18 (18)
	Cytoplasm H score Median (25th percentile, 75th percentile)	0 (0, 0)	0 (0, 0)
	Nuclei Total Detected (H score > 0)	119 (100)	102 (100)
	Nuclei H score Median (25th percentile, 75th percentile)	100 (70, 160)	110 (75, 160)

^a^ The denominator for the percentage calculation is the number of randomized patients with a tumor sample for which an evaluable result was obtained. Percentages are rounded to the nearest integer.

**Table 2:** Summary of baseline tissue and plasma characteristics by isocitrate dehydrogenase 1 (IDH1) mutation status.

Parameter	IDH1 Positive (*n* = 8)	IDH1 Negative (*n* = 108)	*p*-Value
Tissue CD3^+^ Parenchymal infiltrate, *n* (%)	≤1%, 7 (88)	≤1%, 47 (44)	0.026
	2–4%, 0	2–4%, 40 (38)	
	≥5%, 1 (13)	≥5%, 19 (18)	
Tissue CD3^+^ Perivascular infiltrate, *n* (%)	None, 3 (38)	None, 21 (20)	0.0267
	Slight, 5 (63)	Slight, 38 (36)	
	Prominent, 0	Prominent, 47 (44)	
Blood FOXP3 (%), *n* Median (range)	*n* = 8	*n* = 101	0.0394
	1.4 (0.3, 3.2)	0.7 (0.1, 2.7)	
Plasma MDC/CCL22 (pg/mL), *n* Median (range)	*n* = 8	*n* = 104	0.2533
	491 (64, 879)	208 (24, 1220)	
Blood neutrophils (GI/L), *n* Median (range)	*n* = 8	*n* = 98	0.1505
	3.50 (2.65, 11.23)	5.53 (2.01, 16.81)	
Blood neutrophil/lymphocyte ratio, *n*, Median (range)	*n* = 8	*n* = 98	0.0938
	2.63 (1.52, 18.11)	5.74 (0.81, 35.75)	
Plasma TGF-β1 (pg/mL), *n* Median (range)	*n* = 8	*n* = 100	0.1027
	2984 (654, 19774)	2031 (25, 11325)	
Blood CD4^+^ (cells/uL), *n* Median (range)	*n* = 8	*n* = 96	0.1714
	602 (108, 659)	309 (30, 1208)	
Blood CD3^+^ (%), *n* Median (range)	*n* = 8	*n* = 101	0.3016
	27.5 (3.1, 42.8)	13.6 (2.6, 75.4)	
Blood lymphocytes (GI/L), *n* Median (range)	*n* = 8	*n* = 98	0.2562
	1.33 (0.57, 1.99)	0.93 (0.22, 2.74)	
Blood CD4^+^/CD8^+^ Ratio, *n* Median (range)	*n* = 8	*n* = 96	0.368
	1.75 (0.40, 2.58)	1.27 (0.29, 6.31)	
Tissue pSMAD2 cytoplasm H score, *n* (%)	H = 0, 6 (75)	H = 0, 85 (83)	0.6334
	H > 0, 2 (25)	H > 0, 18 (18)	
Blood eosinophils (GI/L), *n* Median (range)	*n* = 8	*n* = 98	0.7284
	0.06 (0.00, 0.16)	0.05 (0.00, 0.23)	
Tissue pSMAD2 nucleus H score, *n* (%)	H ≤ 100, 4 (50)	H ≤ 100, 50 (49)	>0.9999
	H > 100, 4 (50)	H > 100, 53 (52)	

Tissue samples are obtained up to five years prior to plasma/blood sampling; thus temporal changes in plasma/blood sampling may confound results. The median (range) is presented for continuous variables. *p*-values are calculated by analysis of variance using log transformed data for continuous measures and Fisher’s exact test for categorical measures.

## Supplementary Materials

Supplementary materials can be found at www.mdpi.com/1422-0067/18/5/995/s1.
