# Supplementary material for: Biomarker and Histopathology Evaluation of Patients with Recurrent Glioblastoma Treated with Galunisertib, Lomustine, or the Combination of Galunisertib and Lomustine
Source: Int J Mol Sci. 2017 May 6;18(5):995. doi: 10.3390/ijms18050995 (PMC5454908; doi:10.3390/ijms18050995)
Supplement: Supplementary file 1 [file ijms-18-00995-s001.docx]

Supplementary Material

**Table S1.** Time of tissue sample relative to study enrollment.

| **Time Interval** | ***N* = 127 (%)** |
| --- | --- |
| 0 to <0.5 years post-randomization | 29 (23) |
| <0.5 years prior to randomization | 22 (17) |
| 0.5 to <1 years prior to randomization | 45 (35) |
| 1 to <2 years prior to randomization | 22 (17) |
| 2 to <5 years prior to randomization | 8 (6) |
| ≥5 years prior to randomization | 1 (1) |

**Table S2.** Comparison of pathological diagnosis at study entry to central pathology.

|  | **Pathological Diagnosis at Study Entry** | | | |
| --- | --- | --- | --- | --- |
| **Central Pathological Diagnosis** | **Giant Cell Glioblastoma** | **Glioblastoma** | **Gliosarcoma** | **Other** |
| Giant cell glioblastoma | 0 | 1 | 0 | 0 |
| Glioblastoma | 1 | 113 | 1 | 1 |
| Glioma, astrocytoma | 0 | 3 | 0 | 0 |
| Glioma, gliosarcoma | 0 | 1 | 2 | 0 |
| Glioma, pleomorphic Xanthoastrocytoma | 0 | 1 | 0 | 0 |
| Oligoastrocytoma | 0 | 1 | 0 | 0 |
| Protoplasmic astrocytoma | 0 | 1 | 0 | 0 |
| Not evaluated | 0 | 1 | 0 | 0 |

**Table S3.** Frequency counts indicating relationships among Ki67, mitotic scoring and cellular density.

| **Parameter** | | **Cellular Density** | **Cellular Density** | **Cellular Density** |
| --- | --- | --- | --- | --- |
|  |  | **Low (Like Diffuse Astrocytoma)** | **Medium (Like Classical Glioblastoma)** | **High (Like PNET)** |
| Ki67 | ≤5% | 2 (40%) | 4 (5%) | 0 |
|  | 6–10% | 0 | 24 (30%) | 1 (5%) |
|  | 11–20% | 1 (20%) | 36 (44%) | 6 (30%) |
|  | >20% | 2 (40%) | 17 (21%) | 13 (65%) |
| Mitotic scoring | ≤5 Mitosis | 3 (60%) | 28 (28%) | 0 |
|  | 6–20 Mitosis | 2 (40%) | 54 (55%) | 10 (48%) |
|  | >20 Mitosis | 0 | 17 (17%) | 11 (52%) |
| Parameter | | Mitotic Scoring | Mitotic Scoring | Mitotic Scoring |
|  |  | ≤5 Mitosis | 6–20 Mitosis | >20 Mitosis |
| Ki67 | ≤5% | 5 (21%) | 0 | 0 |
|  | 6–10% | 11 (46%) | 14 (25%) | 0 |
|  | 11–20% | 8 (33%) | 28 (51%) | 7 (27%) |
|  | >20% | 0 | 13 (24%) | 19 (73%) |

Percentages use column totals as denominators.

**Table S4a.** Summary of baseline tissue and plasma characteristics by CD3^+^ in tumors.

| **Parameter** | **CD3^+^ Parenchymal Infiltrate, ≤1%** | **CD3^+^ Parenchymal Infiltrate, 2–4%** | **CD3^+^ Parenchymal Infiltrate, ≥5%** | ***p*-Value** |
| --- | --- | --- | --- | --- |
|  | **(*N* = 54)** | **(*N* = 41)** | **(*N* = 20)** |  |
| Blood CD3^+^ (%),  *n* Median (range) | *n* = 50 | *n* = 40 | *n* = 18 | 0.6912 |
|  | 16.4 (2.6, 43.3) | 12.4 (3.4, 75.4) | 14.0 (3.5, 41.5) |  |
| Lymphocytes (GI/L),  *n* Median (range) | *n* = 50 | *n* = 36 | *n* = 19 | 0.8149 |
|  | 1.00 (0.22, 2.74) | 0.89 (0.35, 2.74) | 0.96 (0.24, 2.08) |  |
|  |  |  |  |  |
| Plasma MDC/CCL22 (pg/mL),  *n* Median (range) | *n* = 50 | *n* = 40 | *n* = 19 | 0.4141 |
|  | 288 (24, 1220) | 214 (31, 783) | 183 (41, 572) |  |
|  |  |  |  |  |
| Plasma TGF-β1 (pg/mL),  *n* Median (range) | *n* = 50 | *n* = 39 | *n* = 18 | 0.3905 |
|  | 2068 (25, 19774) | 2574 (25, 5798) | 1527 (25, 3480) |  |
|  | CD3^+^ | CD3^+^ Perivascular Infiltrate, Slight | CD3^+^ Perivascular Infiltrate, Prominent | *p*-value |
|  | Perivascular Infiltrate, None | (*N* = 43) | (*N* = 48) |  |
|  | (*N* = 24) |  |  |  |
| Blood CD3^+^ (%),  *n* Median (range) | *n* = 21 | *n* = 42 | *n* = 45 | 0.5809 |
|  | 16.8 (2.8, 36.1) | 12.4 (2.6, 48.0) | 15.9 (3.5, 75.4) |  |
| Lymphocytes (GI/L),  *n* Median (range) | *n* = 20 | *n* = 41 | *n* = 44 | 0.0675 |
|  | 0.97 (0.34, 1.99) | 0.82 (0.22, 2.04) | 1.08 (0.24, 2.74) |  |
|  |  |  |  |  |
| Plasma MDC/CCL22 (pg/mL),  *n* Median (range) | *n* = 22 | *n* = 40 | *n* = 47 | 0.8503 |
|  | 230 (24, 790) | 227 (31, 1220) | 205 (40, 783) |  |
|  |  |  |  |  |
| Plasma TGF-β1 (pg/mL),  *n* Median (range) | *n* = 21 | *n* = 42 | *n* = 44 | 0.8337 |
|  | 2021 (25, 11325) | 2186 (25, 19774) | 2043 (25, 5798) |  |

*p*-values are calculated by analysis of variance using log transformed data.

**Table S4b.** Summary of baseline tissue and plasma characteristics by pSMAD2 in tumors.

| **Parameter** | **pSMAD2 Cytoplasm,  H > 0 (*N* = 22)** | **pSMAD2 Cytoplasm,  H = 0 (*N* = 97)** | ***p*-Value** |
| --- | --- | --- | --- |
| Plasma CD4^+^CD25^+^CD127^−^/LOFOXp3^+^(cells/uL), *n* | *n* = 21 | *n* = 84 | 0.9364 |
| Median (range) | 16.0 (2.0, 45.0) | 14.5 (2.0, 71.0) |  |
| Blood FOXP3 (%), *n* | *n* = 21 | *n* = 89 | 0.8868 |
| Median (range) | 0.80 (0.10, 1.50) | 0.80 (0.10, 3.20) |  |
| Plasma TGF-β1 (pg/mL), *n* | *n* = 20 | *n* = 90 | 0.5454 |
| Median (range) | 1963 (25, 5306) | 2153 (25, 19774) |  |
|  | pSMAD2 Nuclei,  H > 100 (*N* = 59) | pSMAD2 Nuclei,  H ≤ 100 (*N* = 60) | *p*-value |
| Plasma CD4^+^CD25^+^CD127^−^/LOFOXp3^+^(cells/uL), *n* | *n* = 49 | *n* = 56 | 0.2979 |
| Median (range) | 16.0 (2.0, 71.0) | 14.0 (2.0, 64.0) |  |
| Blood FOXP3 (%), *n* | *n* = 53 | *n* = 57 | 0.2105 |
| Median (range) | 0.80 (0.20, 3.20) | 0.70 (0.10, 2.30) |  |
| Plasma TGF-β1 (pg/mL), *n* | *n* = 53 | *n* = 57 | 0.3703 |
| Median (range) | 2114 (25, 19774) | 2121 (25, 11325) |  |

*p*-values are calculated by analysis of variance using log transformed data.
